# High-Throughput Massively Parallel Sequencing for Fetal Aneuploidy Detection from Maternal Plasma

**DOI:** 10.1371/journal.pone.0057381

**Published:** 2013-03-06

**Authors:** Taylor J. Jensen, Tricia Zwiefelhofer, Roger C. Tim, Željko Džakula, Sung K. Kim, Amin R. Mazloom, Zhanyang Zhu, John Tynan, Tim Lu, Graham McLennan, Glenn E. Palomaki, Jacob A. Canick, Paul Oeth, Cosmin Deciu, Dirk van den Boom, Mathias Ehrich

**Affiliations:** 1 Research and Development, Sequenom Center for Molecular Medicine, San Diego, California, United States of America; 2 Research and Development, Sequenom Inc., San Diego, California, United States of America; 3 Women and Infants Hospital, Alpert Medical School of Brown University, Providence, Rhode Island, United States of America; Tel Aviv University, Israel

## Abstract

**Background:**

Circulating cell-free (ccf) fetal DNA comprises 3–20% of all the cell-free DNA present in maternal plasma. Numerous research and clinical studies have described the analysis of ccf DNA using next generation sequencing for the detection of fetal aneuploidies with high sensitivity and specificity. We sought to extend the utility of this approach by assessing semi-automated library preparation, higher sample multiplexing during sequencing, and improved bioinformatic tools to enable a higher throughput, more efficient assay while maintaining or improving clinical performance.

**Methods:**

Whole blood (10mL) was collected from pregnant female donors and plasma separated using centrifugation. Ccf DNA was extracted using column-based methods. Libraries were prepared using an optimized semi-automated library preparation method and sequenced on an Illumina HiSeq2000 sequencer in a 12-plex format. Z-scores were calculated for affected chromosomes using a robust method after normalization and genomic segment filtering. Classification was based upon a standard normal transformed cutoff value of z = 3 for chromosome 21 and z = 3.95 for chromosomes 18 and 13.

**Results:**

Two parallel assay development studies using a total of more than 1900 ccf DNA samples were performed to evaluate the technical feasibility of automating library preparation and increasing the sample multiplexing level. These processes were subsequently combined and a study of 1587 samples was completed to verify the stability of the process-optimized assay. Finally, an unblinded clinical evaluation of 1269 euploid and aneuploid samples utilizing this high-throughput assay coupled to improved bioinformatic procedures was performed. We were able to correctly detect all aneuploid cases with extremely low false positive rates of 0.09%, <0.01%, and 0.08% for trisomies 21, 18, and 13, respectively.

**Conclusions:**

These data suggest that the developed laboratory methods in concert with improved bioinformatic approaches enable higher sample throughput while maintaining high classification accuracy.

## Introduction

Since the discovery that fetal DNA comprises 3–20% of circulating cell-free (ccf) DNA in maternal plasma [1]–[3], the utilization of ccf DNA as an analyte for diagnostic purposes has been increasingly recognized as a powerful noninvasive alternative for aneuploidy detection during pregnancy. Multiple research and clinical studies have described the performance of massively parallel sequencing (MPS) to detect fetal aneuploidies, most notably trisomies of chromosomes 21, 18, and 13 [4]–[8]. To date, the two largest studies in this field have analysed more than 1500 samples each [7]–[9]. While these studies demonstrated excellent clinical performance with sensitivity and specificity greater than 99%, the underlying sequencing concepts used differ significantly in the depth and width of genomic sampling. One approach uses genome-wide sequencing, while the other uses an approach that limits the analysis to a set of specific genomic regions. The greatest advantage of a whole genome approach is that it enables the impartial analysis of the entire genome, enabling detection of genomic aberrations without *a priori* region selection. Region-specific methods theoretically support higher throughput while still achieving acceptable performance for the two most common trisomies (trisomy 21 and trisomy 18); however, these targeted assays, including those not based on Massively Parallel Sequencing (MPS) [10]–[12], are restricted by the significant amount of re-development required when additional content, for example less frequent trisomies or sex chromosome aneuploidies, is introduced. Additionally, it remains to be seen how well these targeted methods can identify events such as partial trisomies or other large copy number variations.

An ideal method for noninvasive aneuploidy detection using ccf DNA would combine the breadth of information from genome-wide analysis with the throughput advantages of targeted methods. Next generation sequencing technologies are still rapidly evolving and current developments are already improving genome-wide analysis to the point where throughput advantages of targeted methods may become minimal. Here we present the implementation of a set of recent process enhancements that led to a three-fold increase in throughput and a 4-fold reduction in hands-on time while maintaining clinical accuracy. The three main changes of this modified assay include: automated sequencing library preparation; higher multiplexing levels (from 4-plex to 12-plex); and the implementation of new bioinformatic methods. We outline the development process, which comprised >1500 samples, and show the results from a separate internal study analyzing 1269 samples. The results confirm that the new protocol yields a much simplified workflow amenable to higher throughput while maintaining high sensitivity and specificity for the detection of trisomies 21, 18 and 13.

## Materials and Methods

### Sample Acquisition and Blood Processing

Clinical samples for the initial evaluation of the high-throughput assay (library preparation development and assay verification) were collected under three separate Investigational Review Board (IRB) approved clinical protocols (BioMed IRB 301-01, Western IRB 20091396, and Compass IRB 00462). All subjects provided written informed consent prior to undergoing any study related procedures including venipuncture for the collection of up to 20 mL of whole blood into EDTA-K2 spray-dried 10 mL Vacutainers (EDTA tubes; Becton Dickinson, Franklin Lakes, NJ) and 30 mL of whole blood into Cell-Free DNA BCT 10 mL tubes (BCT tubes; Streck, Omaha, NE). Samples collected in EDTA tubes were refrigerated or stored on wet ice and were processed to plasma within 6 hours of the blood draw. Samples collected in BCT tubes were stored at ambient temperature and processed to plasma within 72 hours of the blood draw. The maternal whole blood in EDTA tubes was centrifuged (Eppendorf 5810R plus swing out rotor), chilled (4°C) at 2500 g for 10 minutes, and the plasma was collected. The EDTA plasma was centrifuged a second time (Eppendorf 5810R plus fixed angle rotor) at 4°C at 15,500 g for 10 minutes. After the second spin, the EDTA plasma was removed from the pellet that formed at the bottom of the tube and distributed into 4 mL barcoded plasma aliquots and immediately stored frozen at ≤−70°C until DNA extraction. The maternal whole blood in BCT tubes was centrifuged (Eppendorf 5810R plus swing out rotor), warmed (25°C) at 1600 g for 15 minutes and the plasma was collected. The BCT plasma was centrifuged a second time (Eppendorf 5810R plus swing out rotor) at 25°C at 2,500 g for 10 minutes. After the second spin, the BCT plasma was removed from the pellet that formed at the bottom of the tube and distributed into 4 mL barcoded plasma aliquots and immediately stored frozen at ≤−70°C until DNA extraction.

Clinical samples for multiplexing development and clinical evaluation were collected as previously described [7], [8]. Briefly, whole blood was collected from enrolled subjects prior to an invasive diagnostic procedure. All samples were collected from pregnant females at increased risk for fetal aneuploidy in their first or second gestational trimester as part of an international collaboration (ClinicalTrials.gov NCT00877292). IRB approval (or equivalent) was obtained for this collaboration at each of 27 collection sites. Some data from this study has been previously published [7], [8], [13]. Specifically, data generated in 4-plex format and used herein for the 4-plex to 12-plex comparison has been previously published [7], [8], [13]; however, all data from 12-plex sequencing was generated using the same libraries now sequenced independently in 12-plex format. In addition, for independent confirmation of the high-throughput method, a plasma aliquot from each of the 1269 subjects was processed. Each of these subjects contributed a distinct plasma aliquot to the previously published studies and the fetal karyotype was known. Only samples from singleton pregnancies confirmed to be simple trisomies 21, 18, and 13, or from euploid controls were used.

### Nucleic Acid Extraction

ccf DNA was extracted from maternal plasma using the QIAamp Circulating Nucleic Acid Kit (Qiagen) as previously described [5], [8].

### Fetal Quantifier Assay

The quantity of ccf DNA was assessed for each sample by the Fetal Quantifier Assay (FQA) as previously described [3], [5], [8].

### Semi-Automated Sequencing Library Preparation

Extracted ccf DNA (40 µL) was used as the template for all library preparation. Libraries for the initial increased (12-plex) multiplex experimentation were prepared using previously described methods [7], [8], [13]. Briefly, ccf DNA was extracted and sequencing libraries prepared using oligonucleotides (Illumina), enzymes (Enzymatics), and manual purification processes between each enzymatic reaction using column-based methods (Qiagen). All newly created libraries used in this study were created in 96-well plate format using a modified version of the manufacturer’s protocol for TruSeq library preparation (Illumina) and a semi-automated process that utilized liquid handler instrumentation (Caliper Zephyr; Caliper LifeSciences) with a magnetic bead-based (AMPure XP; Beckman Coulter) cleanup step after the end repair, ligation, and PCR biochemical processes. Since ccf DNA has been well characterized to exist in maternal plasma within a small range of fragment sizes, no size selection was performed upon either the extracted ccf DNA or the prepared libraries [14]. Evaluation of library size distribution and quantification was performed as previously described [8].

### Massively Parallel Sequencing

Twelve isomolar sequencing libraries were pooled and sequenced together on the same lane (12-plex) of an Illumina v3 flowcell on an Illumina HiSeq2000 sequencer. Sequencing by synthesis was performed for 36 cycles followed by 7 cycles to read each sample index.

### Sequencing Control Samples

Sequencing libraries were prepared from pooled ccf DNA isolated from the plasma of two adult male donors diagnosed with trisomy 21 or non-pregnant euploid female donors. Libraries were quantified and mixed at two concentrations (4% trisomy 21 and 13% trisomy 21) to approximate the contribution of ccf fetal DNA in maternal plasma. Library performance was tested prior to the implementation of these controls in the clinical evaluation study.

### Data Analysis

All BCL (base call) output files from the HiSeq2000 were converted to FASTQ format and aligned to the February 2009 build of the human genome (hg19). Since the libraries for multiplex development were prepared manually with the previous version of biochemistry, analysis methods for these data and the GCRM analysis of the 1269 sample evaluation study were applied as previously described [7]. For the high-throughput assay configuration, reads were aligned to hg19 allowing for only perfect matches within the seed sequence using Bowtie 2 [15]. For analysis purposes, the reads mapped to each chromosome were quantified using standard histograms comprising adjacent, non-overlapping 50 kbp long genomic segments. After binning, selection of included 50 kbp genomic segments was determined using a previously described cross validation method [16]. Regions were excluded from further analysis based upon exhibiting high inter-sample variance, low mappability [17], or high percentage of repetitive elements (Repeat Library 20090604; http://www.repeatmasker.org). Finally, aligned reads corresponding to the remaining 50 kbp genomic segments were normalized to account for GC bias [18] and used to calculate the fraction of aligned reads derived from each chromosome. A robust z-score was calculated as previously described using the formula Z_Chromosome_ = (Chromosome Fraction_Sample_ – Median Chromosome Fraction_Flowcell_)/Median Absolute Deviation_Population_. The median chromosome fraction was calculated specific to each flowcell while the Median Absolute Deviation (MAD) was a constant value derived from a static MAD.

## Results

Previous clinical studies using genome-wide MPS for noninvasive fetal aneuploidy detection have shown a range of 92–100% detection rate while maintaining a false positive rate of less than 1%. Our goal was to maintain or improve upon this performance while streamlining the protocol and increasing sample throughput [5], [7], [8]. Improvements focused on three aspects: I) optimizing library preparation to enable robust yield and increased throughput; II) increasing the number of individually molecularly indexed samples pooled together in a single flowcell lane (multiplex level); and III) improving analytical methods for aneuploidy classification.

**Figure 1:**
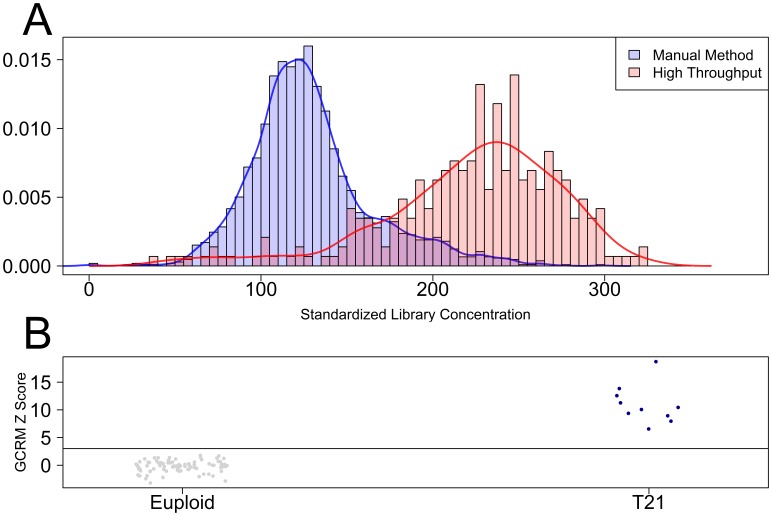
Library preparation optimization. A) The standardized library concentration was compared between semi-automated (n = 287) and manual library preparation methods. B) GCRM based z-scores are shown for each of 93 samples. Confirmed euploid samples (n = 83) are shown in gray; confirmed trisomy 21 samples (n = 10) are shown in blue.

Traditional sequencing library preparation is labor intensive, time consuming and sensitive to operator-to-operator variability. To alleviate these issues, we developed a semi-automated process utilizing a 96-channel liquid handling platform. TruSeq library preparation biochemistry was optimized for the low abundance of ccf DNA recovered from 4 mL of plasma (10–20 ng), which was a 50-fold reduction from the 1 µg recommended input quantity for the TruSeq library preparation kit. In addition, manual purification procedures were replaced with an automated AMPure XP bead purification process optimized for speed, reproducibility and ccf DNA recovery. Comparison of a set of 287 libraries prepared using this method to libraries produced using the manual method [7], [8] revealed an increase in median library concentration from 124 to 225 nM after standardization for elution volume (Figure 1A). The combined semi-automated process produced 96 libraries in 5 hours, requiring only a single technician and 1.5 hours of hands-on labor time. This resulted in a 4-fold increase in throughput coincident with a 4-fold decrease in labor without sacrificing library yield or quality [7], [8]. We sequenced and analyzed 93 libraries (83 confirmed euploid samples and 10 confirmed trisomy 21 samples; Table 1) prepared using this method and found accurate classification performance in this small data set (Figure 1B; Table 2).

**Table 1:** Summary of sample types utilized for each of the studies performed.

	Number of Samples By Karyotype				
Study Description	Unknown	Euploid	Trisomy 21	Trisomy 13	Trisomy 18
Library Optimization	0	83	10	0	0
12-plex Sequencing	0	1629	205	12	54
Verification	1587	1093	134	6	36

**Table 2:** Summary of analysis results for each of the studies performed. Sens = sensitivity.

	Analysis Results By Chromosome						
Study Description	Spec Chr21	Sens Chr21	Spec Chr13	Sens Chr13	Spec Chr 18	Sens Chr18	Analysis Method
Library Optimization	100	100	NA	NA	NA	NA	GCRM
12-plex Sequencing	100	99.5	99.84	91.7	99.73	100	GCRM
Verification	99.91	100	99.92	100	100	100	New

Spec = specificity; NA = Not applicable.

**Figure 2:**
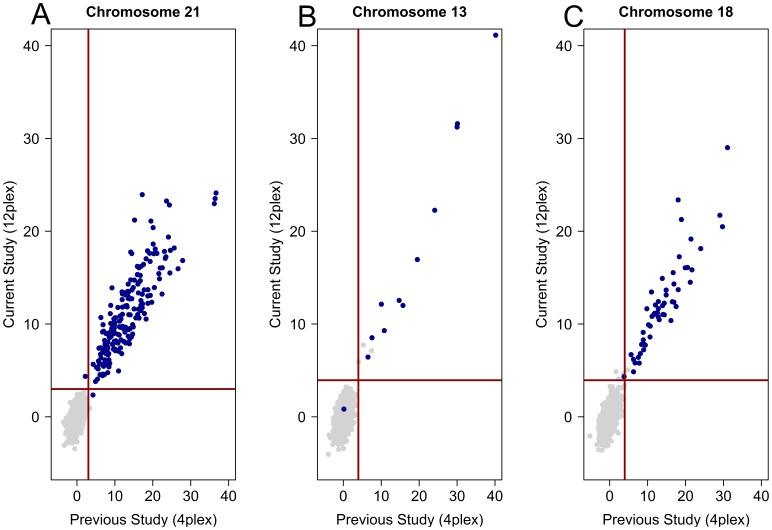
Paired comparison of z-scores. Z-scores were calculated for paired samples with previously described GC normalized, repeat masked z-scores on the x-axis [7] and z-scores from the same libraries sequenced in 12-plex on the y-axis. Samples classified by karyotype analysis as trisomies for A) Chromosome 21, B) Chromosome 13, or C) Chromosome 18 are shown in blue; unaffected samples for each aneuploidy condition are shown in gray. Red horizontal and vertical lines in each plot represent the respective classification cutoff for that chromosome (z = 3 for chromosome 21, z = 3.95 for chromosomes 13 and 18).

Libraries prepared and sequenced in 4-plex during a previous study were sequenced in 12-plex to determine the feasibility of increased multiplexing [7], [8]. Illumina v3 flowcells and sequencing biochemistry, in combination with HCS software improvements, produced a 2.23-fold increase (from 72 to 161 million) in total read counts per lane. We sequenced and analyzed 1900 libraries in 12-plex including 1629 euploid samples, 205 trisomy 21 samples, 54 trisomy 18 samples, and 12 trisomy 13 samples (Table 1) and compared the z-scores for chromosomes 21, 18, and 13 to 4-plex results (Figure 2). Since previous studies had indicated an increase in assay performance using an elevated z-score cutoff, classification was based upon z = 3.95 for chromosomes 18 and 13 [7]. The classification for chromosome 21 remained at z = 3. Using these classification cutoffs, there were a total of 7 discordant classification results between 4-plex and 12-plex sequencing. For chromosome 21, 2 samples previously misclassified (1 false positive, 1 false negative) were correctly classified while a previously noted true positive was not detected. Four samples were misclassified as false positive samples for chromosome 18 whereas they had previously been correctly classified; each of these libraries was highly GC biased. All samples were concordant for trisomy 13 classification. When sequencing in 12-plex, 99.3% of aneuploid samples (204/205 trisomy 21, 54/54 trisomy 18, and 11/12 trisomy 13) were detected with a false-positive rate of 0% (0/1695), 0.27% (5/1846) and 0.16% (3/1888) for trisomies 21, 18 and 13, respectively (Table 2). Overall, these data suggest that the performance of the assay when executed with 12-plex multiplexing is similar to previously published results [7], [8].

Next, a verification study was performed using the optimized library preparation method coupled to 12-plex sequencing (high-throughput assay configuration) to ensure process integrity. We analyzed sequencing results from a total of 2856 samples, 1269 of which had a known karyotype. These 1269 clinical samples were comprised of 1093 euploid, 134 trisomy 21, 36 trisomy 18 and 6 trisomy 13 samples (Table 1). Metrics found previously to be critical to assay success were measured (Figure S1). The median fetal DNA fraction for samples was 0.14 (range: 0.04–0.46), similar to that reported in previous studies (Figure S1A) [5], [7], [8]. The median library concentration was 28.21 nM (range: 7.53–42.19 nM), resulting in a total yield similar to previous methods (Figure S1B) [5]. Finally, the median number of aligned autosomal reads per sample was 16,291,390 (range: 8,825,886–35,259,563) (Figure S1C).

Initial comparison of the data generated from the 1269 samples with known fetal karyotype to a distinct plasma aliquot previously sequenced from the same subject revealed a decrease in the discriminatory distance (difference between the 95^th^ percentile of euploid samples and the 5^th^ percentile of trisomy 21 samples) from 4.9 to 3.09 when analyzed using previously established methods which normalized for GC content and removed reads overlapping with repeat regions (GCRM; Figure S2). To mitigate this effect concomitant with decreasing overall analysis time, a new bioinformatic algorithm specific to the high-throughput assay data was developed. This method based calculations for classification upon only those 50 kbp genomic segments with stable representation across individuals. When applied to the same high-throughput data set, the discriminatory distance between euploid and trisomy 21 samples increased to 6.49 (Figure S2). Overall, new bioinformatic approaches result in an increase in discriminatory distance between euploid and trisomy 21 samples relative to previously described methods.

**Figure 3:**
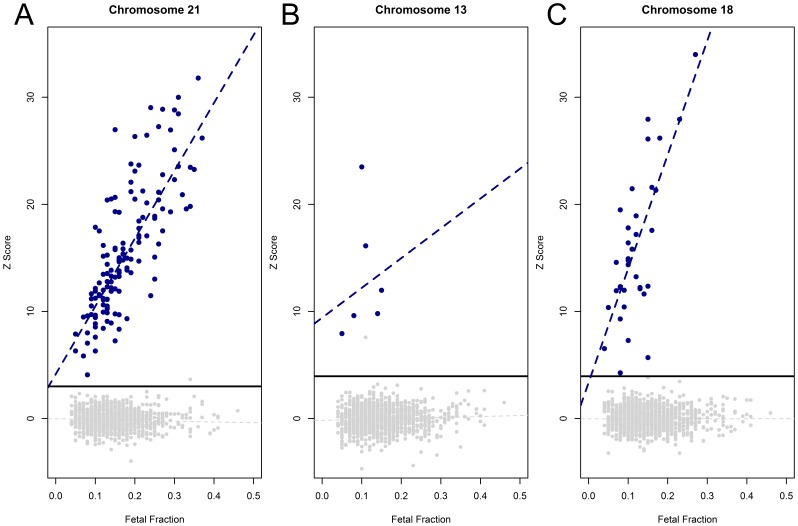
Z-score is linked to fetal fraction. The chromosome specific z-score for each aneuploid chromosome is plotted against the proportion of fetal DNA (fetal fraction). Samples classified by karyotype analysis as trisomies for A) Chromosome 21, B) Chromosome 13, or C) Chromosome 18 are shown in blue; unaffected samples for each aneuploidy condition are shown in gray. Black horizontal line in each plot represents the respective classification cutoff for each chromosome (z = 3 for chromosome 21, z = 3.95 for chromosomes 13 and 18). Dashed blue line in each panel represents a robust linear fit of aneuploid samples. Dashed gray line in each panel represents a robust linear fit of all unaffected samples.

The results from the high-throughput assay were analyzed using the new analysis methods for 67 control and 1269 subject samples. We sequenced 33 libraries prepared from pooled euploid plasma (0% T21 library), 17 control libraries containing 4% trisomy 21 DNA, and 17 control libraries containing 13% trisomy 21 DNA. In all cases, the pooled euploid samples had a z-score less than 3 while the 4% and 13% trisomy 21 control samples had a z-score greater than 3 (Figure S3). We then compared the classification accuracy of the 1269 subject samples with known karyotype information. Based upon these classification limits described above (z-score = 3 for chromosome 21, z-score = 3.95 for chromosomes 18 and 13), all confirmed fetal aneuploidies (134 trisomy 21, 36 trisomy 18, 6 trisomy 13) were detected with a false positive rate of 0.09% (1/1135), <0.01% (0/1233), and 0.08% (1/1263) for trisomies 21, 18, and 13, respectively (Figure 3; Table 2). As previously demonstrated [7], [8], [19], there is a positive correlation between fetal fraction and the magnitude of the z-score while there is no correlation between these metrics for euploid samples.

**Figure 4:**
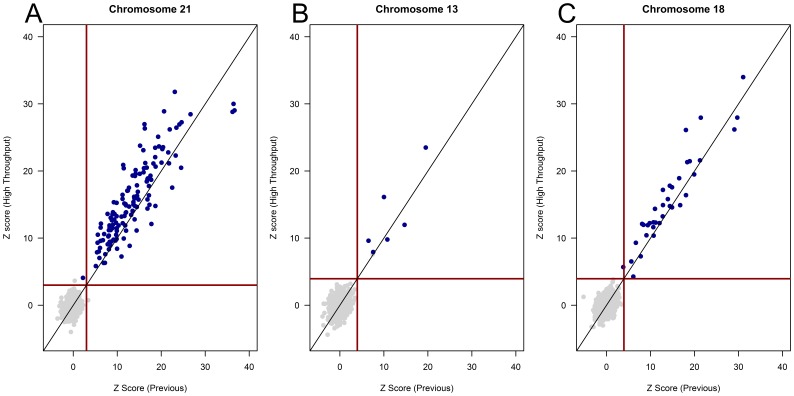
Paired comparison of z-scores. Z-scores were calculated for 1269 paired samples with previously described GC normalized, repeat masked z-scores on the x-axis [7] and z-scores from the high-throughput assay on the y-axis. Samples classified by karyotype analysis as trisomies for A) Chromsome 21, B) Chromosome 13, or C) Chromosome 18 are shown in blue; unaffected samples for each aneuploidy condition are shown in gray. Red horizontal and vertical lines in each plot represent the respective classification cutoff for that chromosome (z = 3 for chromosome 21, z = 3.95 for chromosomes 13 and 18). Black line in plot represents y = x.

Distinct plasma samples from each of the 1269 subjects were previously sequenced and thus serve as a comparison for performance [7], [8]. To ensure a comparable evaluation, z-scores from the previously published studies were calculated using GCRM values and a population size (for median and MAD calculations) of 96 samples, equivalent to the sample number used for median calculations using high-throughput analysis. Comparison of the two studies revealed the correct classification of a previously reported false negative trisomy 21 sample and a previously reported false positive trisomy 21 sample; however, there was one additional false positive during this study (Figure 4). There were no discordant samples when comparing trisomy 13 classification and the correct classification of a single trisomy 18 sample with a previous z-score slightly below 3.95. Evaluation of paired z-scores for aneuploid samples revealed a mean difference of 2.19 for trisomy 21, 1.56 for trisomy 18, and 1.64 for trisomy 13, reflecting an increase in z-score for aneuploid samples using the high-throughput methods. There was a statistically significant increase in z-score for confirmed trisomy 21 and trisomy 18 samples using the high-throughput assay (p = 4.24 e^−12^ and p = 0.0002, respectively; paired wilcox test) relative to the previous study, but no significant difference in z-scores for confirmed trisomy 13 samples (p = 0.31; paired wilcox test). There were no statistically significant differences in chromosome 21, chromosome 18, or chromosome 13 z-scores for euploid samples (p = 0.06, p = 0.90, p = 0.82, respectively; paired wilcox test). This significant increase in aneuploid z-scores without significantly impacting euploid samples further indicates an expansion of the analytical distance between euploid and aneuploid samples for chromosomes 21 and 18 when using the high-throughput assay configuration and new bioinformatic methods.

## Discussion

Several studies, both small and large, have shown the extremely high sensitivity and specificity of fetal aneuploidy detection using MPS-based analysis of ccf DNA [4]–[9], [19]–[25]. Consequently, it is imperative that each new modification to this concept is tested in a sufficiently powered study. The development presented here was preceeded by research activities and followed by additional verification and validation studies conducted in a CLIA-certified laboratory. In total, the entire process of bringing a new laboratory-developed test from research through validation was supported by data from over 5000 tested samples. In this study, we sequenced more than 3400 samples during research, optimization and development. We then performed a clinical evaluation study utilizing 1269 samples, of which we detected all 176 aneuploid samples while maintaining a false positive rate of 0.09% or less for each trisomy.

Next generation sequencing and its implementation into clinical practice is still a rapidly evolving field. In less than one year our throughput per sequencer has more than doubled and the hands-on time required was reduced by 4-fold. Genome-wide analysis methods of ccf DNA will benefit most from this trend. Each advancement in sequencing translates directly into the ability to provide more information to the physician. It has already been shown that the detection of sub-chromosomal alterations as small as 3 MB (such as 22q11.2) is enabled by an approximately 20-fold increase in sequencing [26], [27]. At the current rate of progress, genome-wide analysis of ccf DNA is likely to provide detection of subchromosomal alterrations >10 MB within a year and potentially achieve results comparable to karyotyping within 3 to 5 years.

We have developed an assay which enables a 4-fold increase in library preparation throughput and coupled that to a 3-fold increase in sample multiplexing to allow for high-throughput ccf DNA sample processing. Using these methods in combination with improved analytics, we have shown improved sensitivity and specificity for noninvasive aneuploidy detection while decreasing technician and instrument requirements. Overall, these data suggest that the developed high-throughput assay is technically robust and clinically accurate, enabling detection of all tested fetal aneuploidies (176/176) with a low false positive rate (≤0.09% for each chromosome).

## Supporting Information

Figure S1 **Summary of sequencing paramaters.** A) Proportion of ccf DNA derived from the fetus in 1269 patient samples quantified using methylation-based discriminitory analysis. B) Distribution of the concentration (nM) of prepared sequencing libraries. C) Total number of aligned reads for each of 1269 libraries.
(TIF)

Figure S2 **New bioinformatic methods increase discriminatory distance.** Libraries from two distinct plasma aliquots from the same donor were created, sequenced, and analyzed. Z-scores were calculated as previously described (GCRM) or using the new bioinformatic method (New Method). Blue samples = Trisomy 21, Gray samples = Non-Trisomy 21 (Includes trisomy 13 and trisomy 18 samples). The gap was calculated as the difference in chromosome 21 z-scores from the 95^th^ percentile of non-trisomy 21 samples to the 5^th^ percentile of trisomy 21 samples. GCRM = GC normalized, Repeat Masked.
(TIF)

Figure S3 **Chromosome 21 z-scores of control samples.** Chromosome 21 z-scores of each of three control samples are shown. Euploid pool = distinct library created from pooled euploid plasma; Low Pos = low positive control comprised of mixing 96% non-pregnant female library with 4% trisomy 21 library; High Pos = high positive control comprised of mixing 87% non-pregnant female library with 13% trisomy 21 library. Red line at z = 3 represents classification cutoff for chromosome 21.
(TIF)

## References

[1] Lo YM, Corbetta N, Chamberlain PF, Rai V, Sargent IL, et al (1997) Presence of fetal DNA in maternal plasma and serum. Lancet 350: 485–487. 9274585 10.1016/S0140-6736(97)02174-0

[2] Lo YM, Tein MS, Lau TK, Haines CJ, Leung TN, et al (1998) Quantitative analysis of fetal DNA in maternal plasma and serum: implications for noninvasive prenatal diagnosis. Am J Hum Genet 62: 768–775. 9529358 10.1086/301800 PMC1377040

[3] Nygren AO, Dean J, Jensen TJ, Kruse S, Kwong W, et al (2010) Quantification of fetal DNA by use of methylation-based DNA discrimination. Clin Chem 56: 1627–1635. 20729299 10.1373/clinchem.2010.146290

[4] Chiu RW, Chan KC, Gao Y, Lau VY, Zheng W, et al (2008) Noninvasive prenatal diagnosis of fetal chromosomal aneuploidy by massively parallel genomic sequencing of DNA in maternal plasma. Proc Natl Acad Sci U S A 105: 20458–20463. 19073917 10.1073/pnas.0810641105 PMC2600580

[5] Ehrich M, Deciu C, Zwiefelhofer T, Tynan JA, Cagasan L, et al.. (2011) Noninvasive detection of fetal trisomy 21 by sequencing of DNA in maternal blood: a study in a clinical setting. Am J Obstet Gynecol 204: 205 e1–11. 10.1016/j.ajog.2010.12.060 21310373

[6] Fan HC, Blumenfeld YJ, Chitkara U, Hudgins L, Quake SR (2008) Noninvasive diagnosis of fetal aneuploidy by shotgun sequencing DNA from maternal blood. Proc Natl Acad Sci U S A 105: 16266–16271. 18838674 10.1073/pnas.0808319105 PMC2562413

[7] Palomaki GE, Deciu C, Kloza EM, Lambert-Messerlian GM, Haddow JE, et al (2012) DNA sequencing of maternal plasma reliably identifies trisomy 18 and trisomy 13 as well as Down syndrome: an international collaborative study. Genet Med 14: 296–305. 22281937 10.1038/gim.2011.73 PMC3938175

[8] Palomaki GE, Kloza EM, Lambert-Messerlian GM, Haddow JE, Neveux LM, et al (2011) DNA sequencing of maternal plasma to detect Down syndrome: An international clinical validation study. Genet Med 13: 913–920. 22005709 10.1097/GIM.0b013e3182368a0e

[9] Norton ME, Brar H, Weiss J, Karimi A, Laurent LC, et al.. (2012) Non-Invasive Chromosomal Evaluation (NICE) Study: results of a multicenter prospective cohort study for detection of fetal trisomy 21 and trisomy 18. Am J Obstet Gynecol 207: 137 e1–8. 10.1016/j.ajog.2012.05.021 22742782

[10] Della Ragione F, Mastrovito P, Campanile C, Conti A, Papageorgiou EA, et al (2010) Differential DNA methylation as a tool for noninvasive prenatal diagnosis (NIPD) of X chromosome aneuploidies. J Mol Diagn 12: 797–807. 20847278 10.2353/jmoldx.2010.090199 PMC2963918

[11] Papageorgiou EA, Karagrigoriou A, Tsaliki E, Velissariou V, Carter NP, et al (2011) Fetal-specific DNA methylation ratio permits noninvasive prenatal diagnosis of trisomy 21. Nat Med 17: 510–513. 21378977 10.1038/nm.2312 PMC3977039

[12] Patsalis PC, Tsaliki E, Koumbaris G, Karagrigoriou A, Velissariou V, et al (2012) A new non-invasive prenatal diagnosis of Down syndrome through epigenetic markers and real-time qPCR. Expert Opin Biol Ther 12 Suppl 1S155–161. 22500647 10.1517/14712598.2012.674108

[13] Canick JA, Kloza EM, Lambert-Messerlian GM, Haddow JE, Ehrich M, et al (2012) DNA sequencing of maternal plasma to identify Down syndrome and other trisomies in multiple gestations. Prenat Diagn 32: 730–734. 22585317 10.1002/pd.3892

[14] Lo YM, Chan KC, Sun H, Chen EZ, Jiang P, et al (2010) Maternal plasma DNA sequencing reveals the genome-wide genetic and mutational profile of the fetus. Sci Transl Med 2: 61ra91. 10.1126/scitranslmed.3001720 21148127

[15] Langmead B, Salzberg SL (2012) Fast gapped-read alignment with Bowtie 2. Nat Methods 9: 357–359. 22388286 10.1038/nmeth.1923 PMC3322381

[16] Brunger AT (1992) Free R value: a novel statistical quantity for assessing the accuracy of crystal structures. Nature 355: 472–475. 18481394 10.1038/355472a0

[17] Derrien T, Estelle J, Marco Sola S, Knowles DG, Raineri E, et al (2012) Fast computation and applications of genome mappability. PLoS One 7: e30377. 22276185 10.1371/journal.pone.0030377 PMC3261895

[18] Alkan C, Kidd JM, Marques-Bonet T, Aksay G, Antonacci F, et al (2009) Personalized copy number and segmental duplication maps using next-generation sequencing. Nat Genet 41: 1061–1067. 19718026 10.1038/ng.437 PMC2875196

[19] Sparks AB, Struble CA, Wang ET, Song K, Oliphant A (2012) Noninvasive prenatal detection and selective analysis of cell-free DNA obtained from maternal blood: evaluation for trisomy 21 and trisomy 18. Am J Obstet Gynecol 206: 319 e1–9. 10.1016/j.ajog.2012.01.030 22464072

[20] Ashoor G, Syngelaki A, Wagner M, Birdir C, Nicolaides KH (2012) Chromosome-selective sequencing of maternal plasma cell-free DNA for first-trimester detection of trisomy 21 and trisomy 18. Am J Obstet Gynecol 206: 322 e1–5. 10.1016/j.ajog.2012.01.029 22464073

[21] Chen EZ, Chiu RW, Sun H, Akolekar R, Chan KC, et al (2011) Noninvasive prenatal diagnosis of fetal trisomy 18 and trisomy 13 by maternal plasma DNA sequencing. PLoS One 6: e21791. 21755002 10.1371/journal.pone.0021791 PMC3130771

[22] Chiu RW, Akolekar R, Zheng YW, Leung TY, Sun H, et al (2011) Non-invasive prenatal assessment of trisomy 21 by multiplexed maternal plasma DNA sequencing: large scale validity study. BMJ 342: c7401. 21224326 10.1136/bmj.c7401 PMC3019239

[23] Chiu RW, Sun H, Akolekar R, Clouser C, Lee C, et al (2010) Maternal plasma DNA analysis with massively parallel sequencing by ligation for noninvasive prenatal diagnosis of trisomy 21. Clin Chem 56: 459–463. 20026875 10.1373/clinchem.2009.136507

[24] Faas BH, de Ligt J, Janssen I, Eggink AJ, Wijnberger LD, et al (2012) Non-invasive prenatal diagnosis of fetal aneuploidies using massively parallel sequencing-by-ligation and evidence that cell-free fetal DNA in the maternal plasma originates from cytotrophoblastic cells. Expert Opin Biol Ther 12 Suppl 1S19–26. 22500971 10.1517/14712598.2012.670632

[25] Sparks AB, Wang ET, Struble CA, Barrett W, Stokowski R, et al (2012) Selective analysis of cell-free DNA in maternal blood for evaluation of fetal trisomy. Prenat Diagn 32: 3–9. 22223233 10.1002/pd.2922 PMC3500507

[26] Jensen TJ, Džakula Ž, Deciu C, van den Boom D, Ehrich M (2012) Detection of Microdeletion 22q11.2 in a Fetus by Next-Generation Sequencing of Maternal Plasma. Clin Chem 58: 1148–1151. 22563040 10.1373/clinchem.2011.180794

[27] Peters D, Chu T, Yatsenko SA, Hendrix N, Hogge WA, et al (2011) Noninvasive prenatal diagnosis of a fetal microdeletion syndrome. N Engl J Med 365: 1847–1848. 22070496 10.1056/NEJMc1106975 PMC4308687

