# Supplementary figures and images for: High-Throughput Massively Parallel Sequencing for Fetal Aneuploidy Detection from Maternal Plasma
Source: PLoS One. 2013 Mar 6;8(3):e57381. doi: 10.1371/journal.pone.0057381 (PMC3590217; doi:10.1371/journal.pone.0057381)

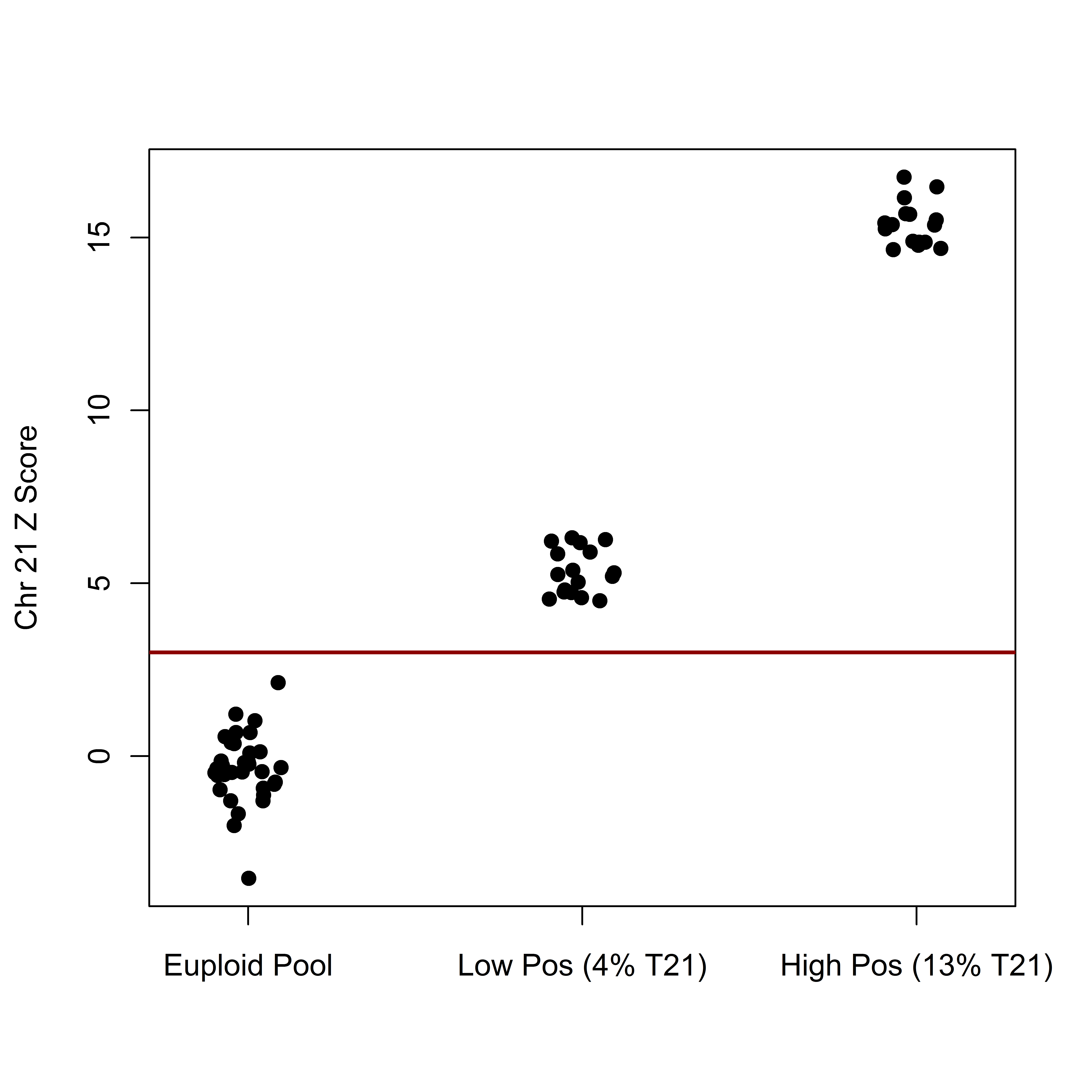

Supplement: Figure S3 — Chromosome 21 z-scores of control samples. Chromosome 21 z-scores of each of three control samples are shown. Euploid pool = distinct library created from pooled euploid plasma; Low Pos = low positive control comprised of mixing 96% non-pregnant female library with 4% trisomy 21 library; High Pos = high positive control comprised of mixing 87% non-pregnant female library with 13% trisomy 21 library. Red line at z = 3 represents classification cutoff for chromosome 21. (TIF) [file pone.0057381.s003.tif]
